# Genetically predicted serum ferritin mediates the association between inflammatory cytokines and non-alcoholic fatty liver disease

**DOI:** 10.3389/fendo.2024.1437999

**Published:** 2024-11-08

**Authors:** XiaoQian Liu, JianHong Jin, BaoFa Wang, LinPu Ge

**Affiliations:** ^1^ Department of Endocrinology, Hangzhou TCM Hospital Affiliated to Zhejiang Chinese Medical University, Hangzhou, Zhejiang, China; ^2^ Department of Orthopedics, The Third Affiliated Hospital of Zhejiang Chinese Medical University, Hangzhou, Zhejiang, China

**Keywords:** inflammatory cytokines, non-alcoholic fatty liver disease, ferritin, metabolism, Mendelian randomization

## Abstract

**Objective:**

Investigating the causal relationship between inflammatory cytokines and Non-alcoholic fatty liver disease(NAFLD) and identifying and quantifying the role of serum ferritin as a potential mediator.

**Methods:**

Genetic summary statistics were derived from open genome-wide association study (GWAS) databases. We conducted a two-sample Mendelian randomization (MR) analysis to investigate the relationship between inflammatory cytokines (8,293 individuals) and NAFLD (8,434 cases, 770,180 controls). Furthermore, we used two-step MR to quantitate the proportion of the effect of serum ferritin-mediated inflammatory cytokines on NAFLD. In this study, we primarily utilized inverse-variance-weighted Mendelian randomization (MR-IVW) and reverse MR analysis methods, while other methods were also performed for sensitivity analysis, false discovery rate (FDR) <0.0012 as statistical significance in MR analyses.

**Results:**

Our results indicated that high levels of Eotaxin, regulated upon activation normal T cell expressed and presumably secreted(RANTES), Interleukin-2(IL-2), macrophage migration inhibitory factor(MIF), tumor necrosis factor-related apoptosis-inducing ligand(TRAIL) and Stem cell factor(SCF) were associated with increased risks of NAFLD, while high Cutaneous T cell-attracting chemokine(CTACK) and Interleukin-16(IL-16) levels that reduced the risk of NAFLD.The proportion of genetically predicted NAFLD mediated by ferritin was 2.1%(95% CI = 1.39%−5.61%).

**Conclusion:**

In conclusion, our study identified a causal relationship between inflammatory cytokines and NAFLD, with a small proportion of the effect mediated by ferritin, but a majority of the effect of inflammatory cytokines on NAFLD remains unclear. Further research is needed on additional risk factors as potential mediators.

## Introduction

NAFLD has emerged as a significant global health issue in recent years (1). Affecting an estimated 25% of adults worldwide, NAFLD is characterized by the accumulation of fat in the liver of individuals with minimal or no alcohol consumption (2, 3). The pathogenesis of NAFLD is multifaceted, involving genetics, metabolism, environmental factors, and inflammatory responses (4–6). Inflammation plays a pivotal role in the development and progression of NAFLD, with inflammatory cytokines identified as key mediators in its pathogenesis. These small, secreted proteins, which regulate immune responses, are released by immune cells and can modulate tissue injury and repair in various organs, including the liver (7).

In clinical practice, our team has observed that approximately 20% to 50% of NAFLD patients exhibit increased levels of serum ferritin (8). This phenomenon raises a critical question: besides the direct inflammatory effects caused by inflammatory cytokines, could they also be indirectly involved in the pathogenesis of NAFLD by regulating other biomarkers (9)? Serum ferritin, a protein widely used to assess body iron stores, has also been implicated in the pathogenesis of NAFLD in recent years (10). Disruptions in iron metabolism play a significant role in the pathophysiology of NAFLD, with iron overload potentially leading to increased oxidative stress and subsequent liver damage (11). Furthermore, elevated serum ferritin levels are associated with enhanced activity of inflammatory cytokines, suggesting that serum ferritin may act as a mediator between inflammatory cytokines and NAFLD (12).

Genetic factors play a crucial role in the pathogenesis of NAFLD. Through numerous genome-wide association studies, scientists have identified several genetic loci closely associated with NAFLD risk (13). These genetic variations may increase the risk of developing NAFLD by affecting key physiological processes such as metabolic pathways, inflammatory responses, or iron metabolism. The aim of GWAS is to explore the association between genetic exposure to phenotype and disease outcomes. MR methods use these genetic variations as instrumental variables to assess the causal relationship between specific exposure factors (e.g., blood lipid levels) and disease outcomes (e.g., NAFLD) (14). As genetic variations are randomly allocated at conception, MR methods can reduce the potential for confounding bias and reverse causality that may arise in traditional observational studies. This bias reduction advantage is also applicable to mediation analysis, which investigates whether one factor indirectly affects outcomes by influencing another factor. Therefore, by examining genetically predicted serum ferritin levels, we can gain a new perspective on the relationship between inflammatory cytokines and NAFLD. This approach may reveal how inflammatory cytokines affect an individual’s susceptibility to NAFLD through genetic pathways, offering new insights for the prevention and treatment of NAFLD.

The objective of this study is to explore the role of genetically predicted serum ferritin levels in the linkage between inflammatory cytokines and NAFLD. Through a detailed examination of the correlation between genetic variations and NAFLD, along with the interactions between serum ferritin, inflammatory cytokines, and NAFLD, our goal is to elucidate the molecular pathways at play in this intricate condition and identify potential targets for the development of novel treatment approaches.

## Materials and methods

### Study Design

**Figure 1:**
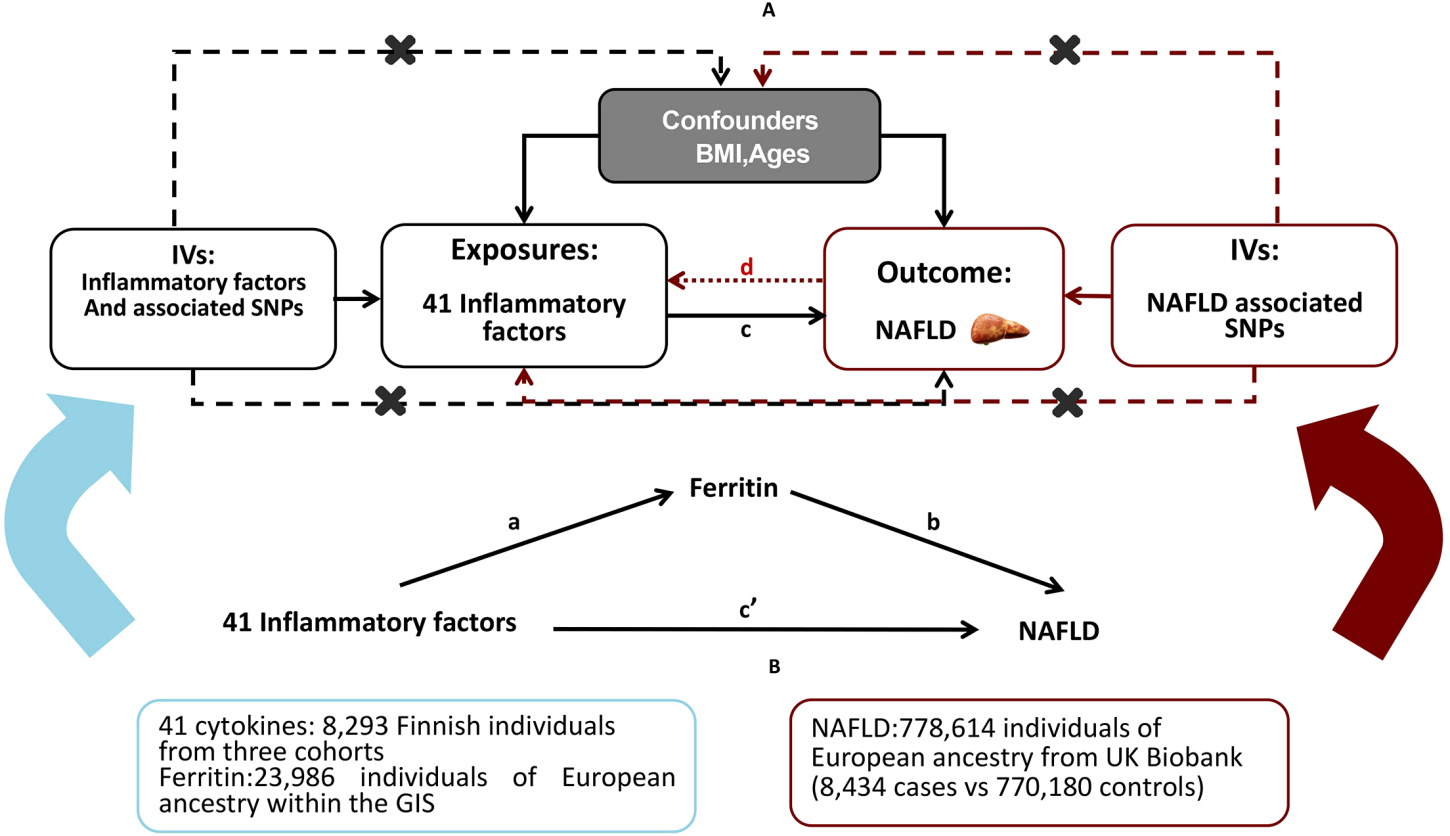
Study overview.

A brief description of the bidirectional MR design displayed in **Figure 1A** The data utilized for our examination are accessible to the public and have received approval from the relevant institutional review boards associated with the individual studies. Consequently, there is no necessity for additional permissions. The outcomes derived from our analysis are comprehensively detailed within the main body of the article as well as its **Supplementary Materials**. The effectiveness of the MR technique hinges on three principal presuppositions: (1) the genetic variant that has been designated as the instrumental variable exhibits a strong correlation with the exposure variable; (2) the genetic variant remains unaffiliated with any potential confounding factors; (3) the influence of genetic variations on the outcome is mediated exclusively through the exposure, precluding any alternative routes of effect (15). In this study, we used summary-level data from published GWASs of 41 systemic inflammatory regulators, serum ferritin and NAFLD. First, we selected genetic variants for serum ferritin and each inflammatory factor. Second, genetic variants associated with NAFLD were exploited to infer the causality from NAFLD to inflammatory factors and serum ferritin. To investigate the potential mediation of ferritin in the causal relationship between inflammatory cytokines and the outcome of NAFLD, we conducted a mediation analysis utilizing a two-step MR framework (**Figure 1B**). The total effect was delineated into an indirect effect mediated by the putative mediator and a direct effect that is independent of the mediator (16). Specifically, the overall impact of inflammatory cytokines on NAFLD was apportioned into two components: 1) the direct effect of inflammatory cytokines on NAFLD(c’ in **Figure 1B**); and 2) the indirect effect conveyed through the mediator ferritin, represented by the product of paths a and b in **Figure 1B**. The proportion of the effect mediated by ferritin was ascertained by dividing the indirect effect by the sum of both direct and indirect effects. Additionally, 95% confidence intervals for the indirect effect were derived using the delta method (17).

### Genetic Instrumental variables for inflammatory factors

The genetic predictors of the 41 systemic inflammatory regulators were obtained from a comprehensive cytokine-related GWAS meta-analysis, which included three independent cohorts. These cohorts consisted of 8,293 Finnish participants from the Cardiovascular Risk in Young Finns Study (YFS) and the ‘FINRISK’ studies (FINRISK1997 and FINRISK2002) (18). Those 41 cytokine distributions were normalized with two-step inverse transformation. An additive genetic model with the adjustment for age, sex, body mass index (BMI), and the first ten genetic principal components.

Genetic variants of ferritin(log10-transformed, ug/l) were obtained from a meta-analysis of GWAS including 23,986 individuals of European ancestry within the Genetics of Iron Status (GIS) consortium (19). Genetic associations between SNPs and ferritin were adjusted for age, principal component scores and other study specific covariates

Summary-level data on NAFLD were extracted from a GWAS of 7786147 individuals (8,437cases vs 770,180 controls) of European ancestry from UK Biobank(2,558 cases vs 395,241 controls) and Estonian Biobank(4,119 cases vs 190,120 controls), adjusted for age, gender, body mass index, genotyping site and the first three ancestry based principal components (20).

Then we employed two-sample MR approaches using GWAS summary statistics to infer the causal association of inflammatory cytokines and serum ferritin with NAFLD. There was no overlapping samples of inflammatory regulators, ferritin, and NAFLD obtained from the different consortiums. The data utilized in this research were sourced from existing literature and publicly accessible databases, following the provision of consent from the participants and adherence to ethical guidelines. Consequently, there is no requirement for additional ethical approval from the institutional review board for this particular study (**Supplementary Table S1**).

To satisfy the MR assumptions, the independence between the selected SNPs was evaluated based on pairwise linkage disequilibrium (21), all SNPs are strongly and independently (R2 < 0.001 within 10 Mb) predicted exposures from the published GWAS at genome wide significance (P <5×10-8). Since only 8 systemic inflammatory regulators and ferritin had 3 or more independent SNPs that reached genome-wide significance and no genome-wide significant SNPs for NAFLD, we adopted a less stringent threshold of 5×10-6 to obtain more SNPs for inflammatory regulators. We evaluated the strength of each SNP using the F statistic, which is a function of the magnitude and precision of the genetic effect on the trait: F = R2(N2)/(1-R2),where R2 is the proportion of the variance of trait explained by the SNP and N is the sample size of the GWAS of SNPs with the trait (22). The R2 values were estimated using the formula R2 = 2×EAF×(1-EAF)×β2, where EAF is the effect allele frequency (EAF) of the SNP and β is the estimated effect of SNP on trait (23). We excluded SNPs with F <10, because F >10 suggested sufficient strength to ensure the validity of the SNPs (24). Moreover, to avoid weak instrumental bias, we evaluated the strength of the IV correlations by calculating the Fstatistic and selected SNPs with F-statistic >10 for inclusion in this MR analysis (25). Finally, whenever unavailable SNPs are found, we use the LDlink Website (https://ldlink.nci.nih.gov/) to search for proxy SNPs (r2 > 0.8) to replace them (26).

### Sensitivity analyses

The IVW method, as a primary approach for estimating the causal relationship between exposure and outcome, calculates the ratio of the effect size of SNPs associated with the outcome to the effect size of SNPs associated with the exposure (27). Sensitivity analyses are essential to ensure the robustness of our conclusions. These analyses primarily include tests for heterogeneity to assess differences among IVs. If substantial differences exist among IVs, it indicates heterogeneity within these IVs. Of particular importance is the assessment for horizontal pleiotropy, where a P-value greater than 0.05 suggests the absence of horizontal pleiotropy. The presence of horizontal pleiotropy renders our conclusions unreliable. Other methods, such as Leave-One-Out (LOO) and Pleiotropy Residual Sum and Outlier (PRESSO), are also used. The LOO test involves iteratively excluding individual observations from the analysis dataset to evaluate their impact on the results. PRESSO is a statistical tool used to detect potential shared genetic effects (pleiotropy) influencing the genotype-outcome relationship (28).

### Statistical analysis

We ensured that the effect of SNPs on exposure and outcome corresponded to the same alleles by harmonising the summary statistics for both datasets. To infer causal associations, we conducted TSMR analyses using multiple methods, including IVW, weighted median regression, MR−Egger regression, simple mode, and weighted mode. The IVW method, as a primary approach for estimating the causal relationship between exposure and outcome, is used to calculate the ratio of the effect size of SNPs associated with the outcome to the effect size of SNPs associated with the exposure (27). The IVW method was used as the primary method for MR, which combined the Wald ratio estimates of different SNPs to provide a consistent estimate of the causal effect of exposure on the outcome (28). The reliability of the IVW method depends on the absence of horizontal pleiotropy of the IVs (29). When at least half of the SNPs were effective IVs, the weighted median method provided a consistent estimate of the causal effect (30). To adjust for multiple testing, we calculated the false discovery rate (FDR) using the Benjamini-Hochberg method. MR−Egger regression was used to confirm the existence of horizontal pleiotropy, and its intercept represented the effect estimate of horizontal pleiotropy (31). Even when the IVs have horizontal pleiotropy, MR−Egger regression can still be used to obtain an unbiased estimation of causal associations. Compared to the MR−Egger method, the weighted median method improved the accuracy of the results (32). Simple mode and weighted mode were used for complementary analyses (33). Additionally, scatter plots and funnel plots were used to demonstrate the robustness of the correlation and lack of heterogeneity. All analyses were conducted in R 4.3.2 software using the R packages TwoSampleMR and MR-PRESSO (34). The R packages randomForest and ggplot2 were used for plotting.

## Results

**Figure 2:**
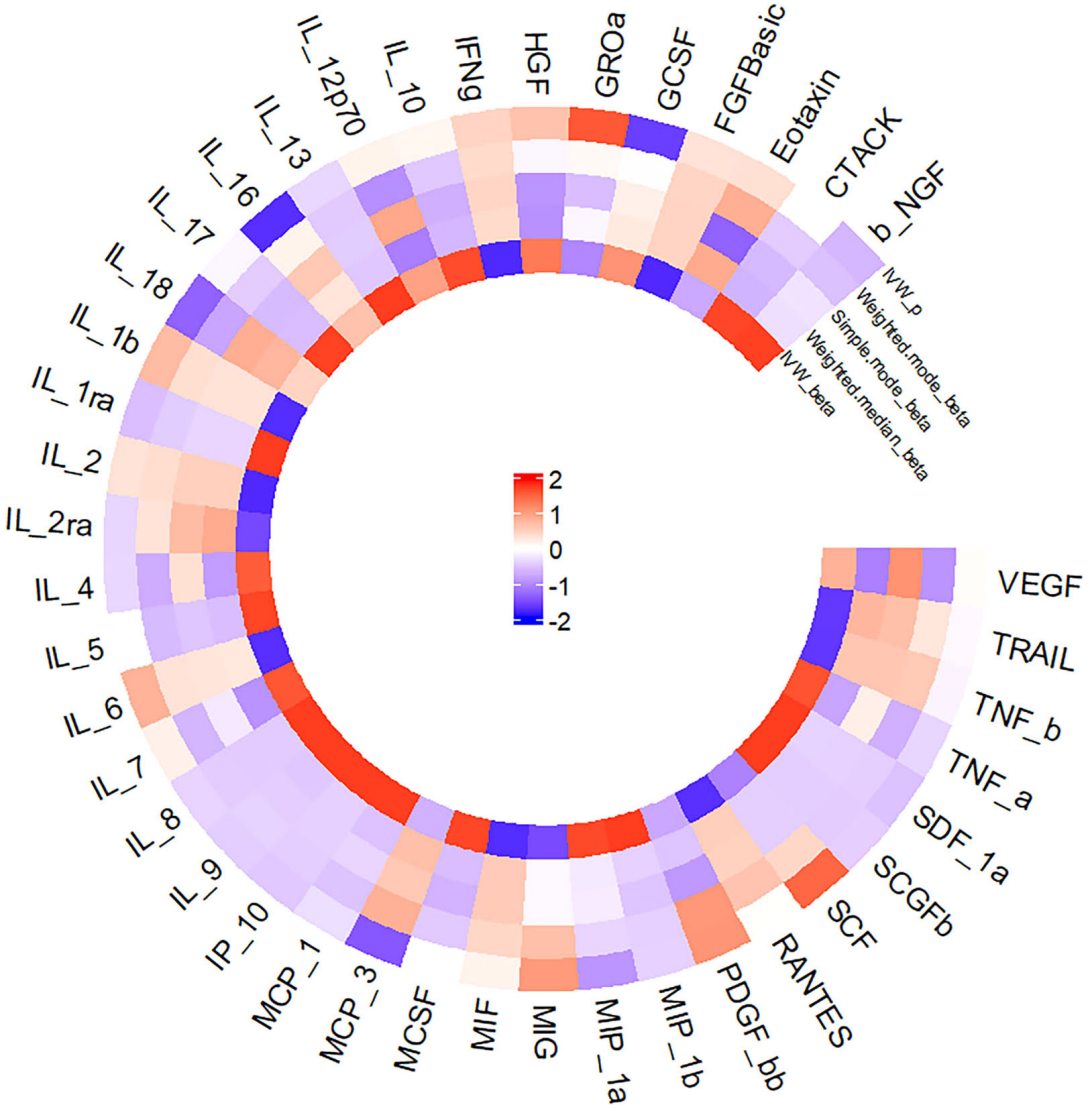
Circular Heat Map.

**Figure 2** presents a circular heat map illustrating the suggestive genetic correlation between inflammatory cytokines and serum ferritin in relation to NAFLD.

### Association of inflammatory cytokines with NAFLD

**Figure 3:**
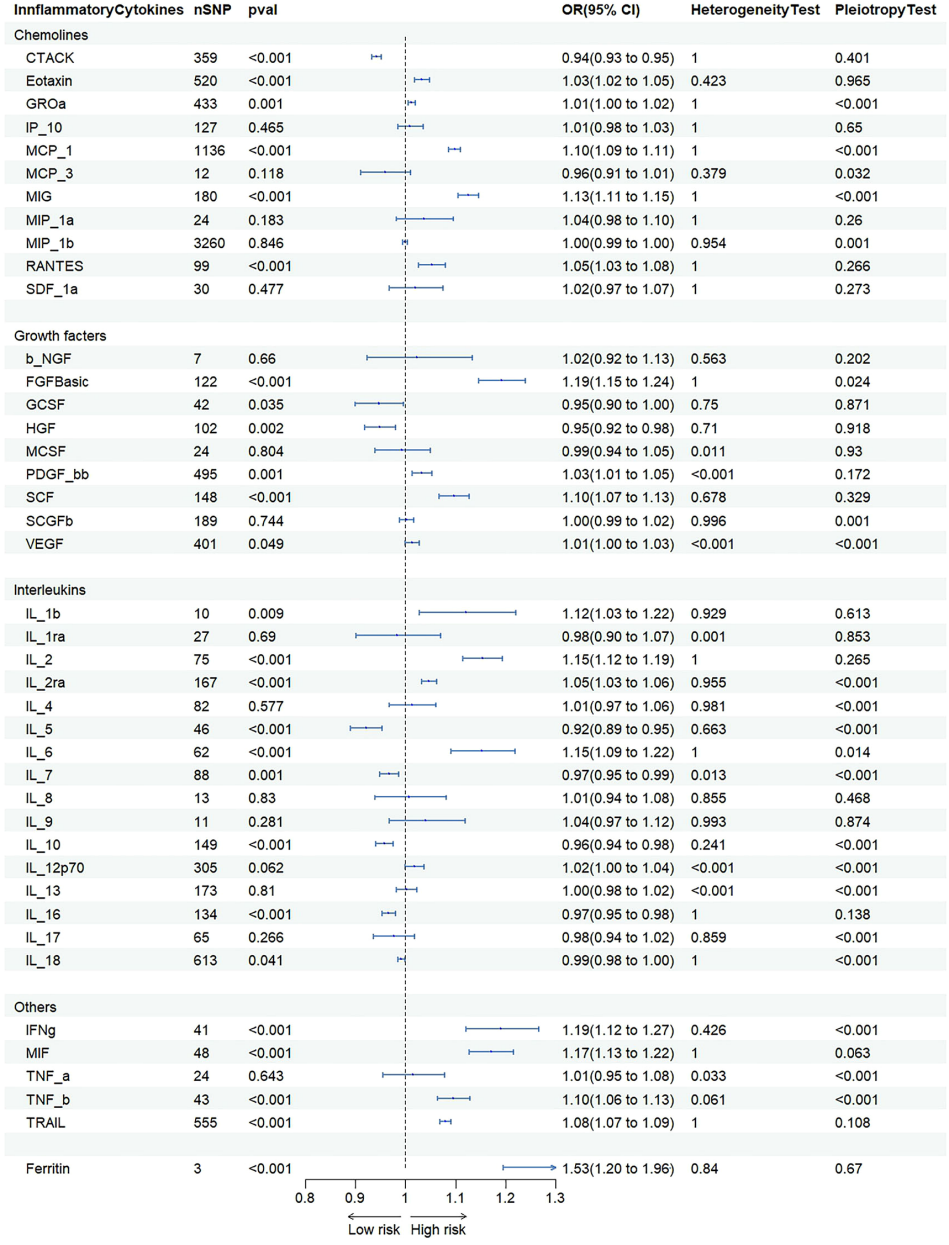
Association of inflammatory cytokines with NAFLD.

IVW were used to estimate the causal relationship between genetically predicted inflammatory cytokines and NAFLD (**Figure 3** and **Supplementary Table S2**). As 41 inflammatory cytokines were used for MR analysis as exposure, Eotaxin(OR=1.033 95%CI=1.018–1.048,p=8.9e-06), IL-2(OR=1.154 95%CI=1.116–1.194, p=1.2e-16), RANTS(OR=1.053 95%CI =1.026–1.80, p=8.0e-05), MIF(OR=1.171 95%CI=1.128–1.216, p=1.8e-16, SCF(OR=1.097 95%C =1.067–1.128, p=9.6e-11) and TRAIL(OR=1.080 95%CI=1.069–1.091, p=8.2e-49) acted as a promote, while CTACK(OR=0.942 95%CI=0.932–0.952, p=1.0e-29) and IL-16(OR=0.966 95%CI=0.953–0.980, p=1.5e-06) acted as an inhibitor.The scatter plot is depicted in **Supplementary Figure S1**. To adjust for multiple testing, we calculated the false discovery rate (FDR) using the Benjamini-Hochberg method. However, the results of our MR analysis showed no reverse causality for genetically predicted NAFLD on inflammatory cytokines by using the IVW method (**Supplementary Table S3**).

### Association of inflammatory cytokines with ferritin

**Figure 4:**
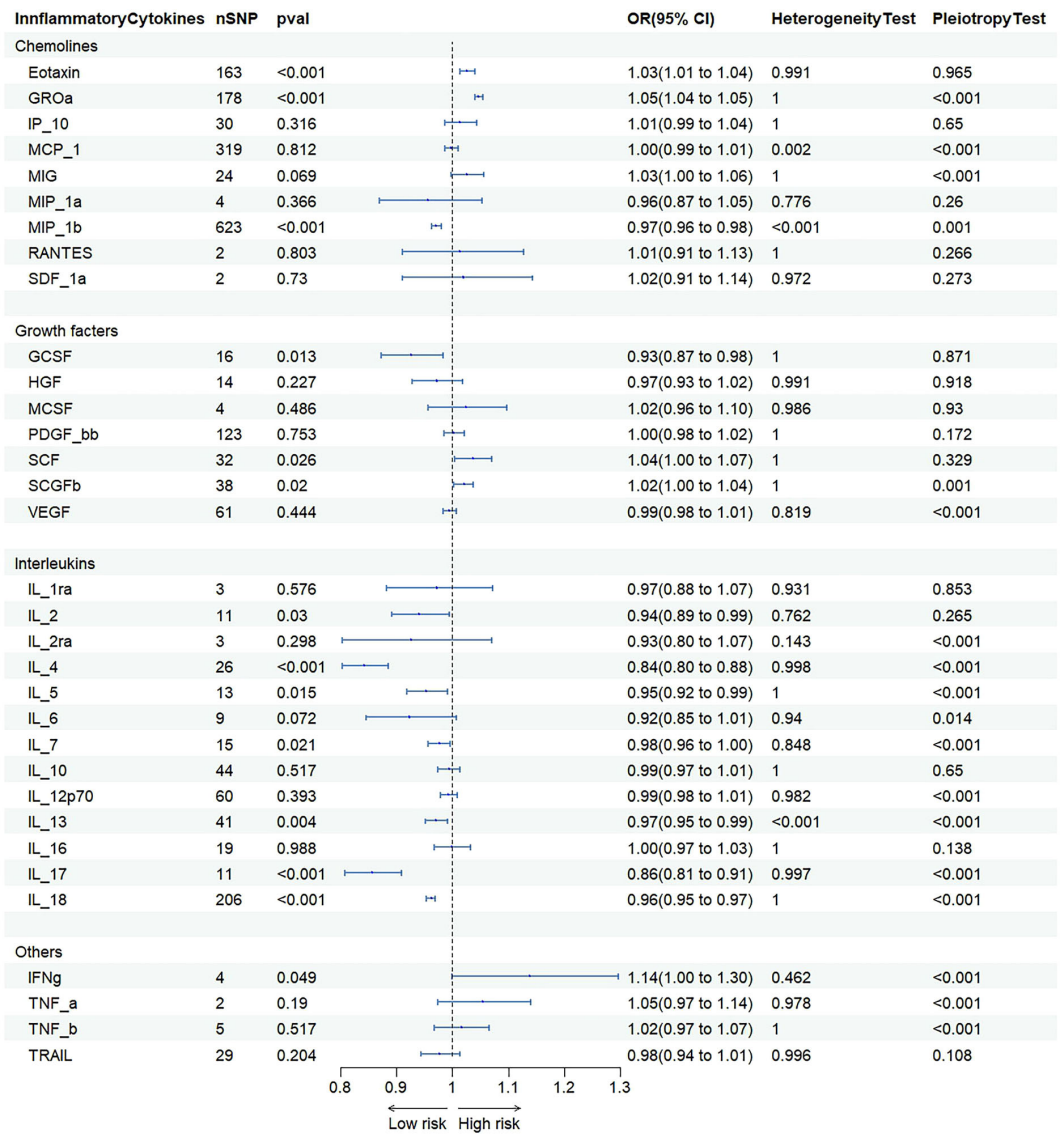
Association of inflammatory cytokines with ferritin.

IVW were used to estimate the causal relationship between genetically predicted inflammatory cytokines and serum ferritin (**Figure 4** and **Supplementary Figure S4**). As 41 inflammatory cytokines were used for MR analysis as exposure, Eotaxin (OR=1.033 95% CI = 1.018–1.048, p = 8.9e-06) acted as a promote, while GCSF (OR=0.942 95% CI = 0.932–0.952, p = 1.0e-29) acted as an inhibitor. The scatter plot is depicted in **Supplementary Figures S6**, **S7**. To adjust for multiple testing, we calculated the false discovery rate (FDR) using the Benjamini-Hochberg method.

### Association of ferritin with NAFLD

Genetic instruments for ferrintin explained 2.2% of its variance, with an F-statistic of 11.4. As shown in **Figure 3** and **Supplementary Table S2**, we presented all genetic instruments associated with ferritin at the genome-wide significance level (P < 5 x 10-8). As shown in **Figure 3**, genetically predicted ferritin was significantly positively correlated with NAFLD [OR=1.53 95% CI=1.20-1.96, p=7.16E-04] by using the IVW method.

### Proportion of the association between inflammatory cytokines and NAFLD mediated by ferritin

We analyzed ferrintin as a mediator of the pathway from inflammatory cytokines to NAFLD. We found that NAFLD was associated with increased Eotaxin, which in turn was associated with an increased risk of NAFLD. As shown in **Figure 4**, our study showed that ferritin accounted for 2.1% of the increased risk of inflammatory cytokine associated with NAFLD (proportion mediated: 2.1%; 95% CI = 1.39%−5.61%).

### Sensitivity analysis

The results of the tests for heterogeneity and pleiotropy when inflammatory cytokines and serum ferritin were used as exposures are presented in **Supplementary Table S2**. The results of the tests for heterogeneity and pleiotropy when inflammatory cytokines were used as outcomes are presented in **Supplementary Table S3**. The results of the tests for heterogeneity and pleiotropy when analyzing the associations of inflammatory cytokines with the risk of serum ferritin are presented in **Supplementary Table S4**. In addition, the scatter plot, funnel plot, forest plot, density plot and leave-one-out analysis results are presented in **Supplementary Figures S1**-**S7**. Details of SNPs analyzing the associations of inflammatory cytokines and serum ferritin with the risk of NAFLD are presented in **Supplementary Tables S5**-**S13**.Details of SNPs analyzing the associations of inflammatory cytokines with the risk of serum ferritin are presented in **Supplementary Tables S14**, **S15**.

## Discussion

NAFLD is a non-bacterial chronic inflammatory state of the liver, characterized by elevated inflammatory markers and potentially progressing to cirrhosis or hepatocellular carcinoma (35). In the context of this condition, we conducted a two-sample Mendelian randomization analysis to explore the relationship between inflammatory cytokines and NAFLD.

Through the PhenoScanner website (http://www.phenoscanner.medschl.cam.ac.uk/), we excluded Single Nucleotide Polymorphisms (SNPs) associated with hepatitis and systemic infections to minimize the impact of confounding factors. Ultimately, after excluding confounding and other factors, the association between certain inflammatory cytokines and NAFLD was identified as a genuine causal effect.

We also utilized MR-Egger regression to assess the potential pleiotropic effects of the selected SNPs as instrumental variables (IVs), which may provide valuable insights into whether horizontal pleiotropy (such as hepatitis, systemic infections, etc.) affects the analysis. Some analyses with horizontal pleiotropy were excluded. This study found that genetically predicted levels of Eotaxin, IL-2, RANTES, MIF, and TRAIL were positively correlated with the risk of NAFLD, while CTACK and IL-16 were negatively correlated. Some of these findings are consistent with the results of other studies that are less likely to be affected by confounding biases and reverse causality.

Eotaxin and RANTES are both members of the CC chemokine family, playing a crucial role in inflammation and immune responses. These factors selectively attract eosinophils and are involved in the recruitment of immune cells to sites of inflammation. In the context of NAFLD, Eotaxin-1 may exacerbate the inflammatory state of the liver by promoting the migration and activation of inflammatory cells (36). RANTES may intensify liver damage by promoting the infiltration of immune cells and enhancing the inflammatory response. For instance, RANTES may be involved in the hepatic inflammatory process by attracting specific immune cell subsets, such as T cells and monocytes (37). Additionally, studies have shown that levels of Eotaxin-1 are associated with metabolic disorders such as insulin resistance, impaired glucose tolerance and abnormal lipid metabolism, which are all risk factors for the development of NAFLD (38). In some studies, the levels of Eotaxin-1 have been found to correlate with the extent of hepatic steatosis and liver enzyme levels (such as alanine aminotransferase and aspartate aminotransferase) (39). Therefore, Eotaxin-1 may serve as a biomarker linking these metabolic disorders with NAFLD. Given the role of Eotaxin-1 in the development of NAFLD, it may represent a potential therapeutic target. For example, the use of anti-Eotaxin-1 monoclonal antibodies may help to alleviate inflammation and immune-mediated liver damage associated with NAFLD.

IL-2 is an immunomodulatory cytokine primarily produced by CD4+ T cells. The regulation of IL-2 gene expression involves various transcription factors and plays a significant role in modulating immune responses and anti-tumor activity (40). Although the direct link between IL-2 and NAFLD has not yet been clearly established, considering the role of IL-2 in immune regulation, it may indirectly affect the development of NAFLD by influencing the hepatic immune microenvironment. For example, IL-2 may influence inflammatory responses and hepatocyte damage by regulating T cell activity in the liver. However, further research is needed to clarify the specific role of IL-2 in NAFLD.

Similarly, TRAIL, known for its ability to induce apoptosis in various cancer cells, has also been found to play a role in regulating inflammatory responses and the progression of liver diseases.

MIF is a cytokine with multiple biological functions and plays a key role in inflammation and immune responses (41). The association between MIF and NAFLD may be related to its role in regulating immune cell function and promoting inflammatory responses. MIF may be involved in the development of NAFLD by affecting the migration and activation of macrophages.

On the other hand, the chemokine CTACK (CCL1) and IL-16 are negatively correlated with NAFLD. CTACK is a skin-related chemokine that controls the migration and aggregation of immune cells, potentially affecting the inflammatory response, some study have confirmed that CTACK has a significant correlation with certain autoimmune liver diseases (42). IL-16 is mainly produced by activated CD8+ T cells, with CD4 as its receptor, and can chemoattract CD4+ T cells, monocytes, and eosinophils, inducing the expression of IL-2R and HLA class II molecules in T cells and monocytes (43). Recent studies have found that IL-16 is present in many cells and plays an important regulatory role in T cell function and intercellular communication (44). IL-16 plays different roles in various pathological processes; it acts as a chemoattractant in some diseases, a pro-inflammatory factor in others, and may even function as an inhibitor in Th2-mediated diseases (Exogenous interleukin-16 inhibits antigen-induced airway hyper-reactivity, eosinophilia, and Th2-type cytokine production in mice) (45). It is speculated that both may exert a protective effect by regulating the activity of immune cells in the hepatic microenvironment, and further research is needed to clarify their specific roles in the pathogenesis of NAFLD.

Furthermore, studies have shown an inseparable link between iron, redox biology, and inflammation. During infection, elevated levels of ferritin represent an important host defense mechanism by depriving bacteria of iron for growth and protecting immune cell function (46). It may also have a protective role by limiting the production of free radicals and mediating immune regulation. Additionally, hyperferritinemia is a key acute-phase reactant, and clinicians use it as an indicator for therapeutic intervention, aiming to control inflammation in high-risk patients (47). One view is that hyperferritinemia is an “innocent bystander” biomarker of uncontrolled inflammation, useful for measuring the effectiveness of interventions (48). Another school of thought suggests that ferritin induction may be a protective negative regulatory loop (47). Some scholars consider ferritin to be a key mediator of immune dysregulation, especially in extreme hyperferritinemia, through direct immunosuppression and pro-inflammatory effects (49). Clearly, further research is needed to determine the role of ferritin as a biomarker and mediator in uncontrolled inflammatory conditions, as its occurrence identifies patients at high risk of mortality, and its resolution can predict an improvement in their survival rate.

Serum ferritin is a common protein associated with reactive oxygen species, leading to necrotic inflammation and fibrosis. In the latest prospective cohort study, the area under the curve(AUC) for diagnosing NAFLD using serum ferritin was 0.791 (50). A study confirmed that elevated serum ferritin greater than 1.5 times the upper limit of normal is associated with the diagnosis of NAFLD and advanced fibrosis (51). Another study established a scoring system combining serum ferritin, type IV collagen 7S, and fasting insulin to predict NAFLD (52).

**Figure 5:**
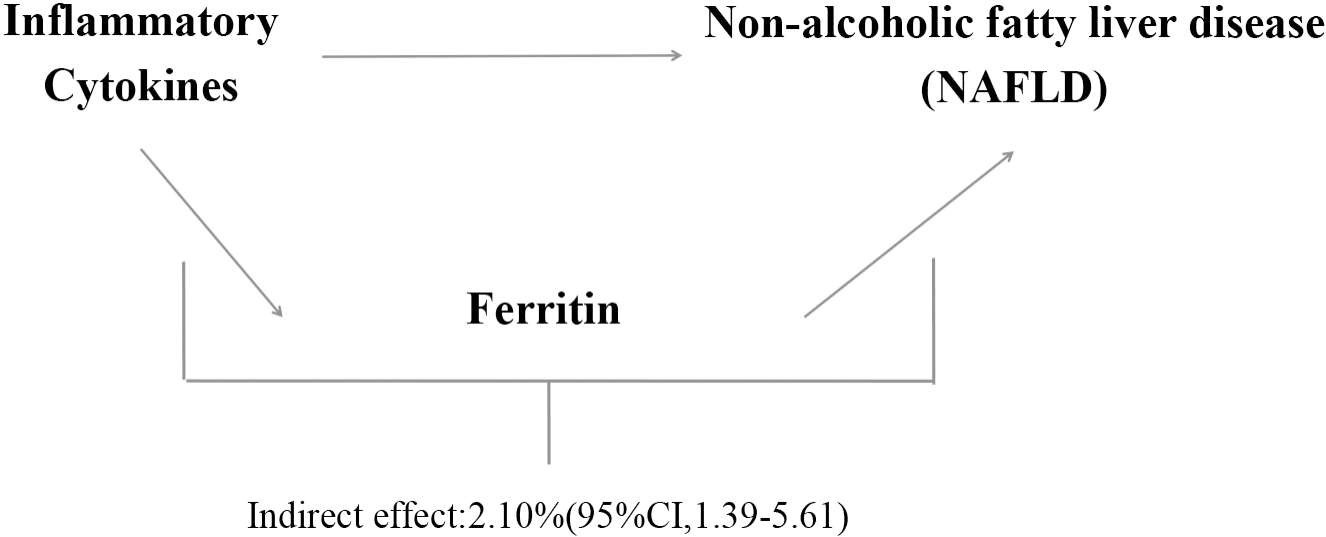
Proportion of the association between inflammatory cytokines and NAFLD mediated by ferritin.

This study suggests that ferritin was significantly positively correlated with NAFLD [OR=1.53, 95% CI, 1.20-1.96; P=7.16E-04] using the IVW method. We analyzed ferritin as a mediator of the pathway from inflammatory cytokines to NAFLD. We found that NAFLD was associated with increased Eotaxin, which in turn was associated with an increased risk of NAFLD. As shown in **Figure 5**, our study showed that ferritin accounted for 2.1% of the increased risk of inflammatory cytokine associated with NAFLD (proportion mediated: 2.1%; 95% CI = 1.39%−5.61%).

Our study has several aspects that make it superior to others: (1) The statistical data for exposure and outcome are summarized from recent GWAS, with a large sample size and no overlapping samples in each study cohort. (2) Strict criteria were set for selecting IVs, enhancing statistical power. (3) Since genetic variations are distributed across multiple chromosomes, the potential for gene-gene interactions to affect the results is likely minimal. (4) To improve the accuracy of the estimates, this study excluded SNPs that may be related to confounding factors and heterogeneity in the studies. This study does have some limitations: (1) The GWAS used only included populations of European ancestry. Therefore, additional studies should be conducted in non-European populations to explore the mediating effects. (2) NAFLD has many subtypes, and further research is needed to analyze whether the subtype results are consistent with our study. (3) Accurately describing the causal link of exposure factors is crucial for the success of mediation analysis, as statistical methods cannot distinguish between the concepts of mediation and confounding. (4) The assessment of inflammatory cytokines was limited. Other types of analyses should be conducted to explore other potential mediating factors.

## Conclusion

In summary, our study attempts to preliminarily establish the certain relationship between certain inflammatory cytokines and NAFLD, with a portion of the effect mediated by ferritin, but the majority of the impact of these inflammatory cytokines on NAFLD is not yet clear. Further research is needed to identify other risk factors that may act as potential mediators. In clinical practice, NAFLD patients with hyperferritinemia warrant closer monitoring.

## Data Availability

The original contributions presented in the study are included in the article/**Supplementary Material**. Further inquiries can be directed to the corresponding author.

## Supplementary material

Supplementary Table 1 Supporting Information **Supplementary Tables S1**-**S15**.

Supplementary Figure 1 Inflammatory Cytokines-NAFLD scatter plot.

Supplementary Figure 2 Inflammatory Cytokines-NAFLD funnel plot.

Supplementary Figure 3 Inflammatory Cytokines-NAFLD forest plot.

Supplementary Figure 4 Inflammatory Cytokines-NAFLD leave-one-out plot.

Supplementary Figure 5 Inflammatory Cytokines-NAFLD density plot.

Supplementary Figure 6 Eotaxin-Ferritin Plot.

Supplementary Figure 7 G-CSF-Ferritin Plot.
