# Supplementary figures and images for: Genetically predicted serum ferritin mediates the association between inflammatory cytokines and non-alcoholic fatty liver disease
Source: Front Endocrinol (Lausanne). 2024 Nov 8;15:1437999. doi: 10.3389/fendo.2024.1437999 (PMC11581845; doi:10.3389/fendo.2024.1437999)

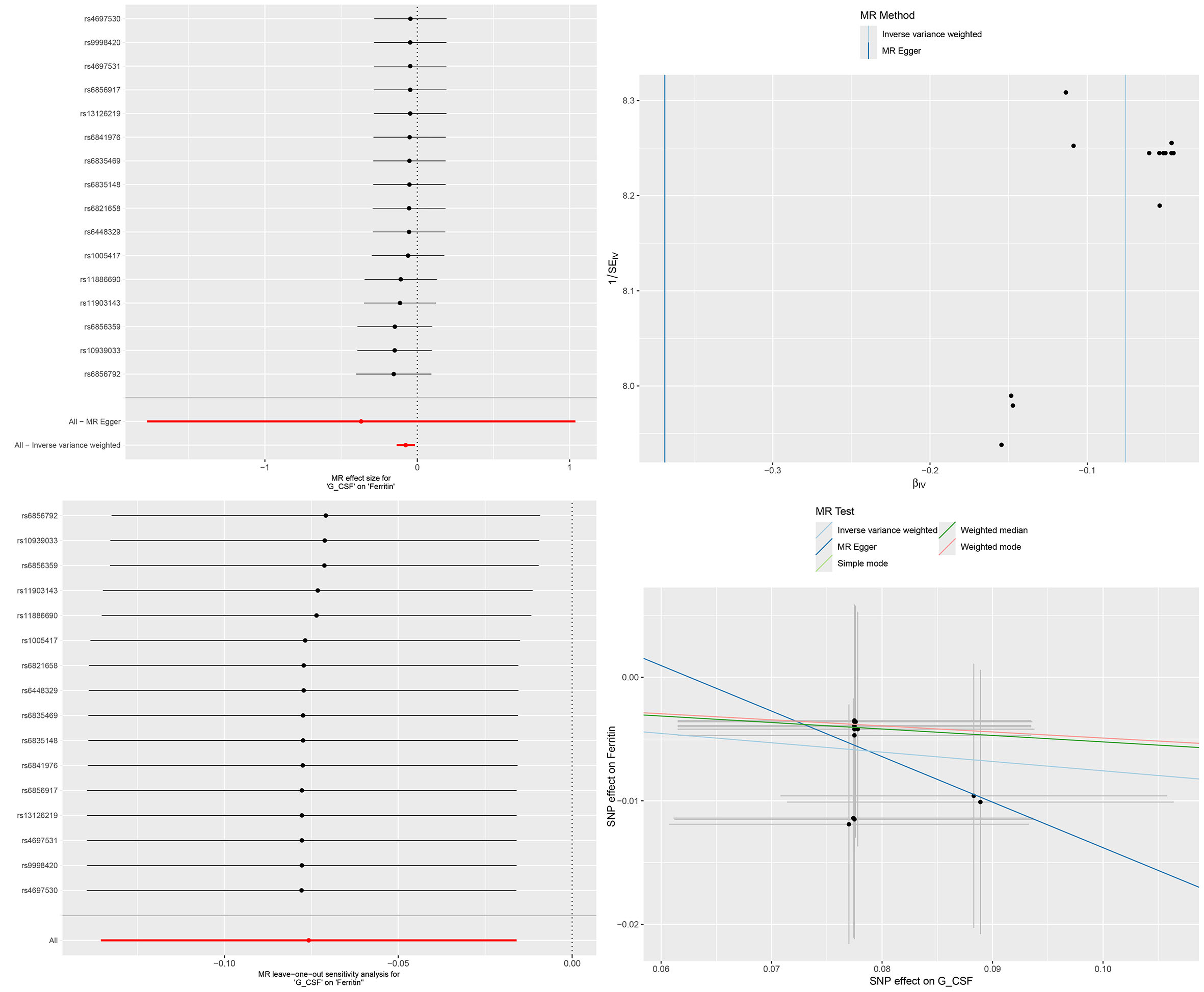

Supplement: Supplementary Figure 1 — Inflammatory Cytokines-NAFLD scatter plot. [file Image1.jpeg]

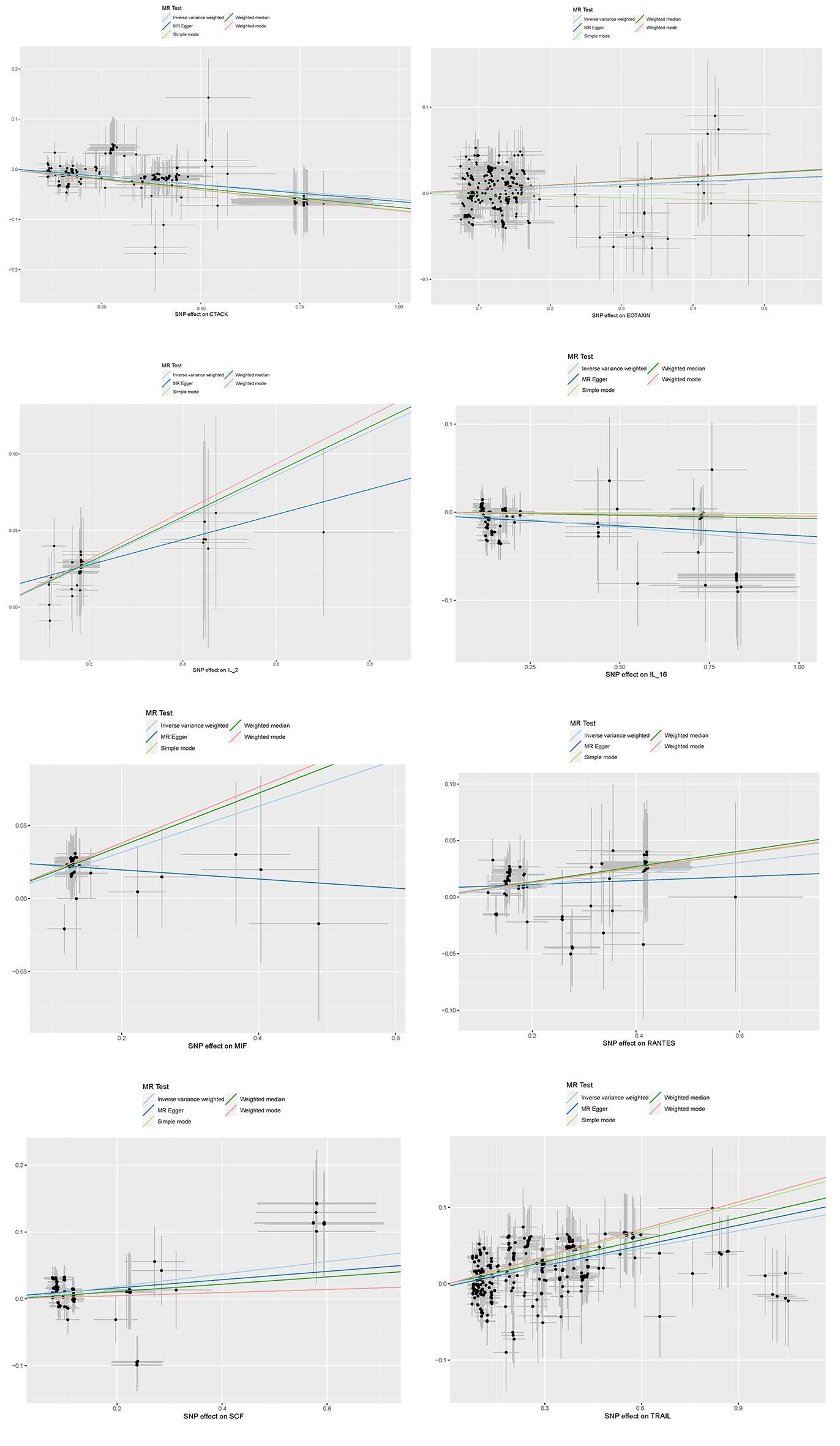

Supplement: Supplementary Figure 2 — Inflammatory Cytokines-NAFLD funnel plot. [file Image2.jpeg]

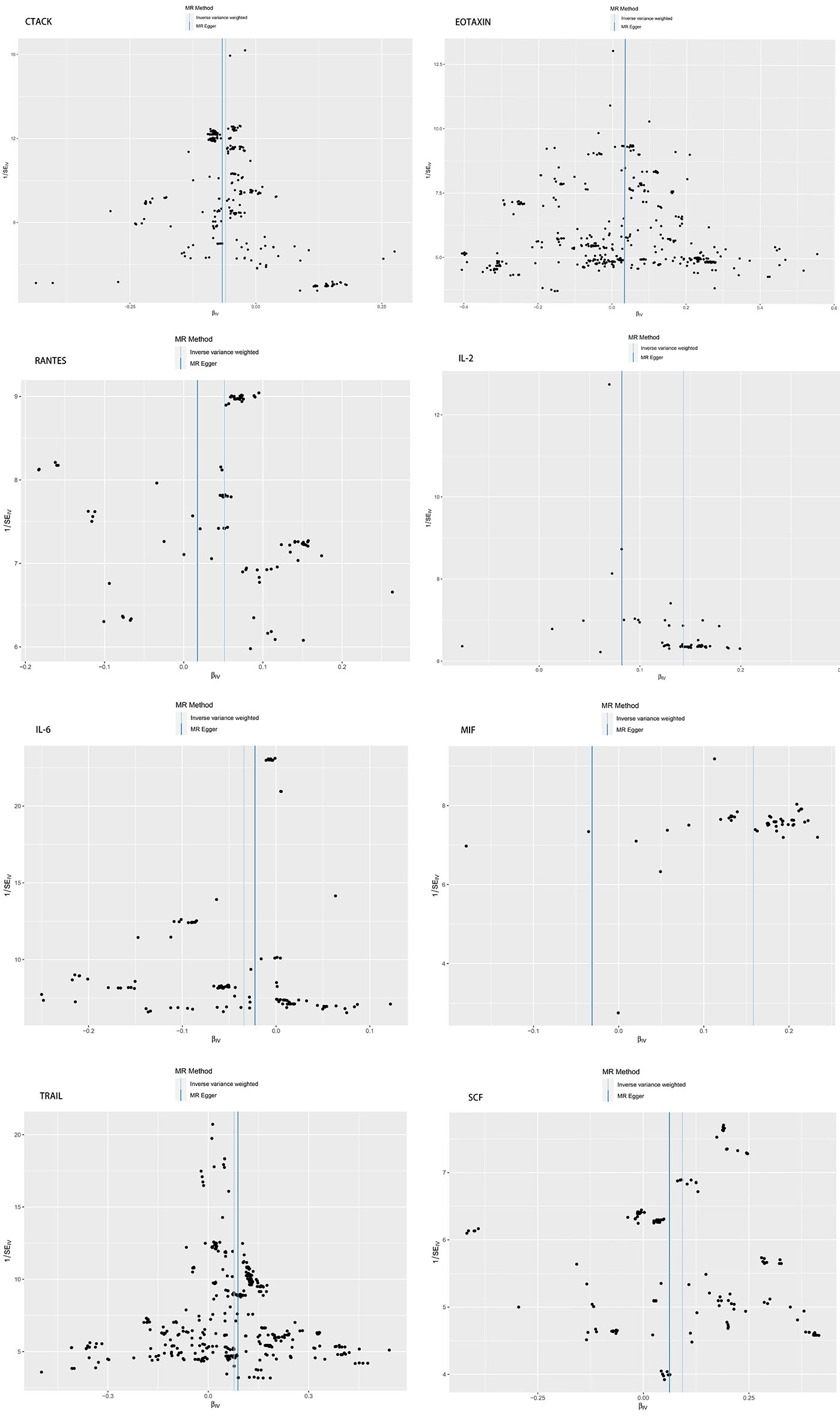

Supplement: Supplementary Figure 3 — Inflammatory Cytokines-NAFLD forest plot. [file Image3.jpeg]

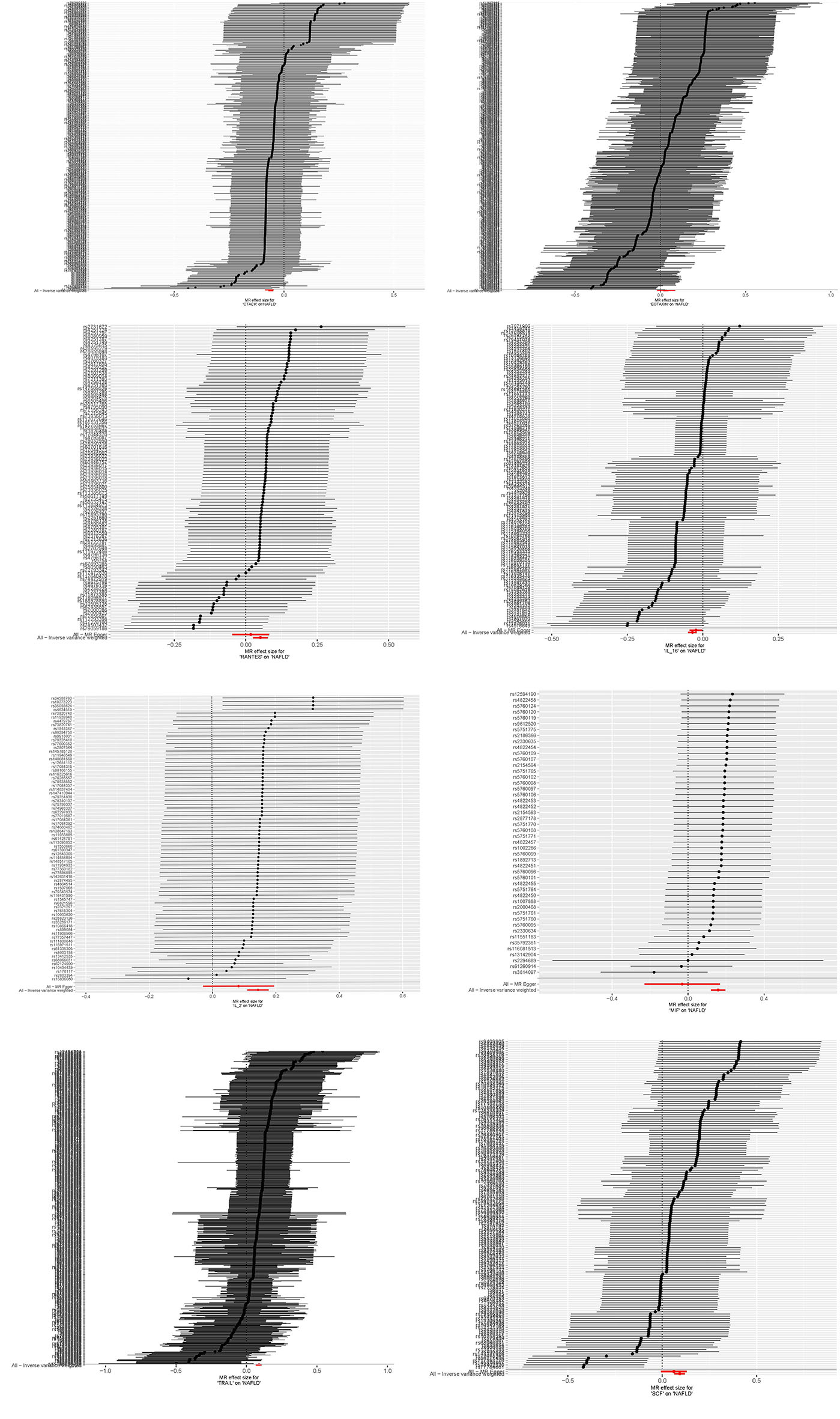

Supplement: Supplementary Figure 4 — Inflammatory Cytokines-NAFLD leave-one-out plot. [file Image4.jpeg]

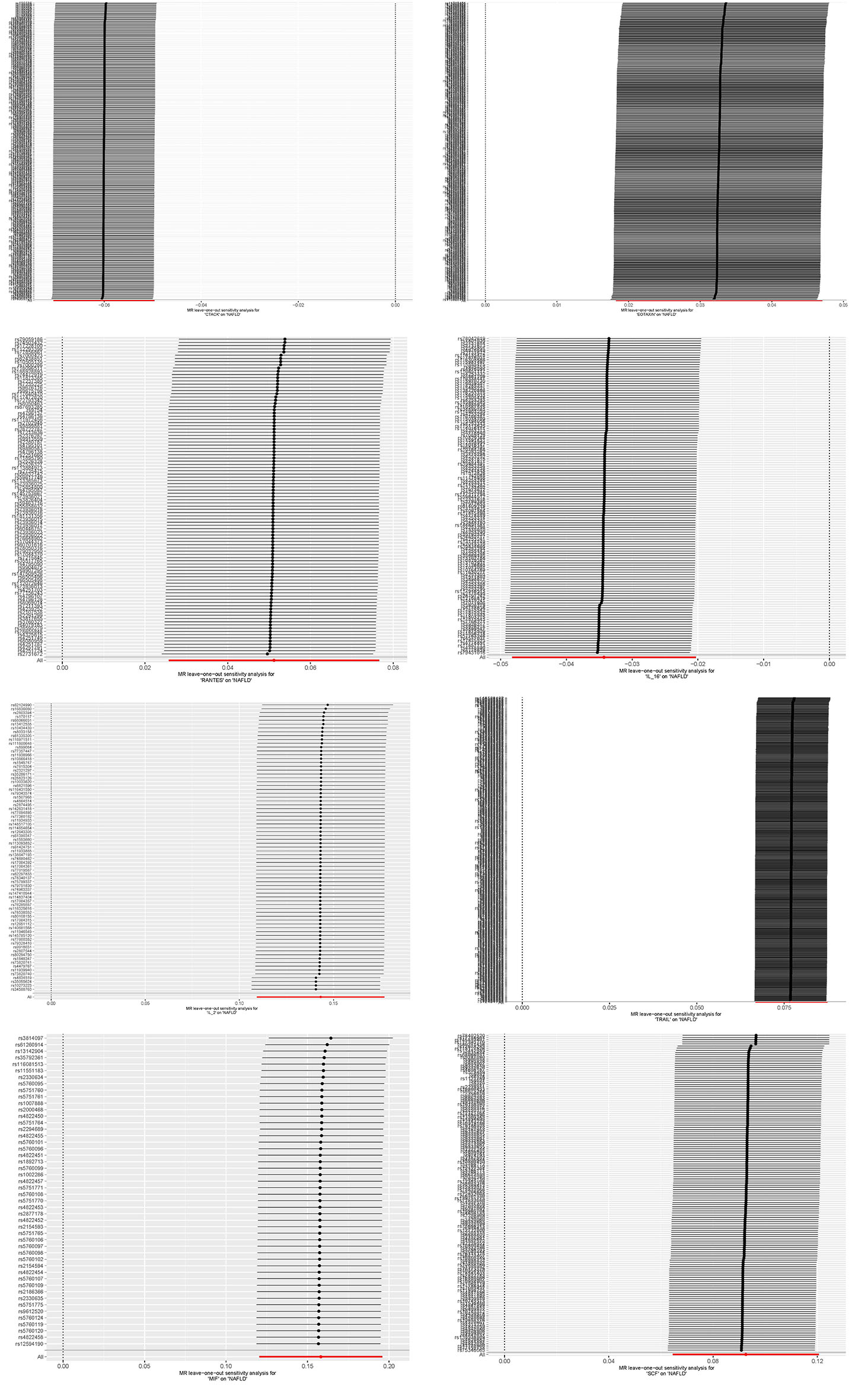

Supplement: Supplementary Figure 5 — Inflammatory Cytokines-NAFLD density plot. [file Image5.jpeg]

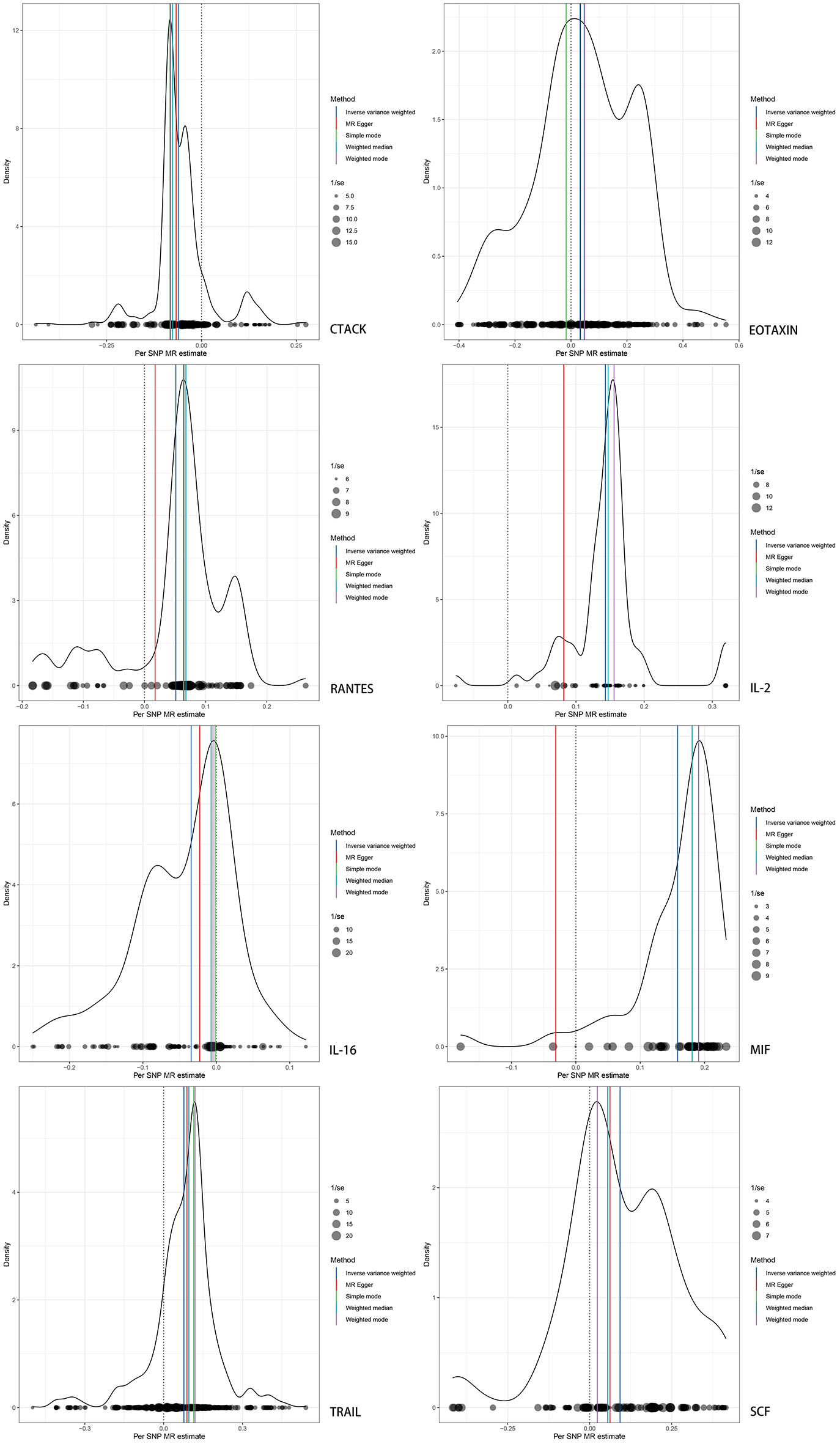

Supplement: Supplementary Figure 6 — Eotaxin-Ferritin Plot. [file Image6.jpeg]

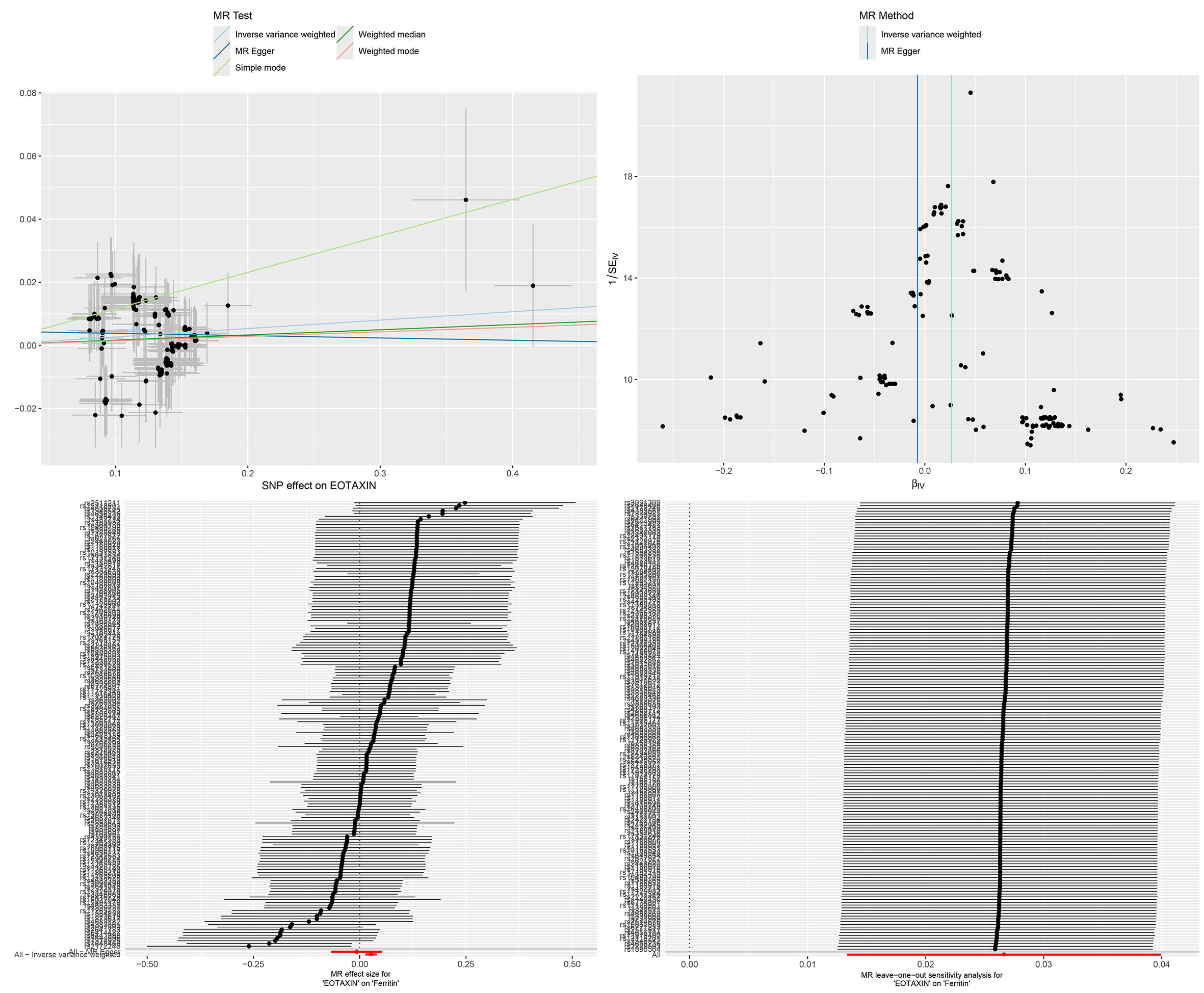

Supplement: Supplementary Figure 7 — G-CSF-Ferritin Plot. [file Image7.jpeg]
